# Inhibition of autophagy via 3-methyladenine alleviates the progression of preeclampsia: 3-Methyladenine alleviates the progression of preeclampsia

**DOI:** 10.3724/abbs.2024096

**Published:** 2024-07-08

**Authors:** Fei Ma, Ning Ding, Lin Xie, Xiangyu Zhao, Shengchao Ma, Guizhong Li, Yinju Hao, Jiantuan Xiong, Kai Wu, Yideng Jiang, Huiping Zhang

**Affiliations:** 1 NHC Key Laboratory of Metabolic Cardiovascular Diseases Research Ningxia Medical University Yinchuan 750004 China; 2 Department of Medical Genetics Maternal and Child Health of Hunan Province Changsha 410008 China; 3 Ningxia Key Laboratory of Vascular Injury and Repair Research Ningxia Medical University Yinchuan 750004 China; 4 School of Basic Medical Sciences Ningxia Medical University Yinchuan 750004 China; 5 Department of Clinical Medicine Ningxia Medical University Yinchuan 750004 China; 6 General Hospital of Ningxia Medical University Yinchuan 750004 China

**Keywords:** preeclampsia, autophagy, RNA sequencing, 3-methyladenine

## Abstract

Autophagy is a cellular mechanism for self-renewal that involves the breakdown of cytoplasmic proteins or organelles within lysosomes. Although preeclampsia (PE) exhibits several characteristics that could imply disrupted autophagy, there is limited evidence supporting the notion that impaired placental autophagy directly causes PE, as indicated by differential expression profiling of whole placental tissue. In this study, we aim to explore the significance of autophagy in maintaining pregnancy and its association with PE. First, the RNA-seq results show that 218 genes are differentially expressed in placentas from preeclamptic pregnancies. Notably, KEGG pathway analysis reveals significant enrichment of genes related to autophagy-related signaling pathways, including the PI3K-Akt signaling pathway, the AMPK signaling pathway, and the mTOR signaling pathway. Additionally, our findings indicate an increase in autophagy in placentas from pregnancies complicated by preeclampsia as well as in trophoblasts subjected to hypoxic conditions. Next, we examine the impact of 3-methyladenine (3-MA), a targeted inhibitor of autophagy, on the progression of PE. The administration of 3-MA profoundly alleviates the severity of PE-like symptoms in rats subjected to reduced uterine perfusion pressure (RUPP). The findings from our study suggest that inhibiting autophagy may serve as a promising approach for adjuvant chemotherapy for PE.

## Introduction

Preeclampsia (PE) is a pregnancy disorder characterized by hypertension that occurs in approximately 5% of pregnancies and ranks among the primary causes of maternal and perinatal mortality worldwide [1]. This condition is marked by the development of high blood pressure after 20 weeks of pregnancy, along with the presence of proteinuria and/or acute kidney injury in the mother. Additionally, it may be accompanied by liver dysfunction, neurological complications, thrombocytopenia or hemolysis, as well as fetal growth restriction [ 2, 3]. Despite extensive research efforts and advancements in clinical management, the underlying mechanisms of PE remain elusive, and no effective treatment options other than delivery are currently available [4].

Autophagy is a highly conserved cellular process observed in organisms ranging from yeast to mammals, and it serves to preserve cellular homeostasis in response to stress [5]. Initially, autophagy was shown to function as a means of generating energy during periods of starvation [6]. Indeed, a wealth of evidence suggests that autophagy has a multitude of functions, including stress protection, energy regulation, immune modulation, cellular differentiation, proliferation, and programmed cell death [ 7‒ 9]. Recent studies have demonstrated an increase in autophagy in placentas affected by complications such as PE or foetal growth restriction [10]. Furthermore, there is a positive correlation between the severity of these conditions and increased formation of autophagosomes, suggesting that abnormal autophagic activity is one of the underlying mechanisms responsible for compromised trophoblast invasion [11]. 3-Methyladenine (3-MA) is a recognized phosphatidylinositol 3-kinase (PI3K) inhibitor and an autophagy modulator [12]. 3-MA can reportedly induce cell death in cancer cells through both autophagy-dependent and autophagy-independent pathways [13]. Nonetheless, comprehensive research investigating the potential of 3-MA as an inhibitor of PE progression is scarce.

In this study, we used 3-MA, an inhibitor of autophagy, to explore the effect of autophagy during PE. This study provides the first evidence for the role of autophagy in PE.

## Materials and Methods

### Patients and study samples

Approval for this study was granted by the Clinical Research Ethics Committee of Ningxia Medical University (approval No. 2017-083), and informed consent was obtained from all participants in accordance with ethical guidelines. The samples included in this study were obtained from women who underwent cesarean delivery at the General Hospital of Ningxia Medical University between 2017 and 2021. A total of 45 preeclamptic pregnancies were included in this study and were selected based on the clinical and pathological criteria established by the American College of Obstetrics and Gynecology. The control group consisted of 40 placentas obtained from healthy pregnant patients (PCs) with no history of medical illness or medication use. The placental tissues were retrieved within 5 min postdelivery from four specific positions in the mid-section, situated between the chorionic and maternal basal surfaces. Immediately after retrieval, the tissues were thoroughly rinsed with PBS buffer and then carefully preserved at ‒80°C.

### Cell culture

The JEG-3 cell line (derived from human choriocarcinoma) and HTR8/SVneo cell line (normal trophoblast) were acquired from the IBS Cell Center of Fudan University (Shanghai, China). These cells were cultured in RPMI-1640 or Ham’s F-12 media supplemented with 10% fetal bovine serum (FBS), 100 U/mL of penicillin and 100 mg/L of streptomycin (Solarbio, Beijing, China). The cell cultures were maintained at 37°C with 5% CO _2_ in a humidified air environment. To simulate severe hypoxic conditions, 1 × 10 ^6^ cells were seeded into a 60-mm dish. After 24 h of incubation, the cells were transferred to a NAPCO Series 8000WJ incubator (Thermo Fisher Scientific, Waltham, USA) set at 1% O _2_ and 5% CO _2_ and maintained at 37°C for 48 h. Each experiment was repeated three times.

### Animal treatment

Adult female Sprague-Dawley (SD) rats (13‒14 weeks old) were procured from Ningxia Medical University Laboratory Animal Center (Yinchuan, China). The rats were allowed unrestricted access to food and water and were housed in a controlled environment with a 12/12-h light/dark cycle in a temperature-controlled room (22‒24°C). Following a one-week acclimatization period, the rats were mated overnight with healthy male SD rats at a ratio of 2:1. The first day of mating was considered gestational day 1, as determined by the presence of vaginal sperm plugs. On gestational day 14, the rats underwent either a sham operation or surgical reduction of uterine perfusion pressure (RUPP) by banding the lower abdominal aorta above the iliac bifurcation and the main uterine branches of the ovarian arteries. The rats were randomly divided into three groups: sham, RUPP, and RUPP treated with 3-MA (15 mg/kg/day; Sigma Aldrich, St Louis, USA). Each group consisted of six rats. On gestational day 20, the rats were humanely euthanized using pentobarbital, and the placentas were subjected to hematoxylin-eosin (HE) staining. The animal experiments were conducted in accordance with the guidelines approved by the Committee on the Ethics of Animal Experiments of Ningxia Medical University (approval No. 2021-250).

### RNA-sequencing (RNA-seq) assay

Total RNA was extracted from placental tissues to construct cDNA libraries. In the case of the small RNA cDNA library, the complete RNA was first ligated with both an RNA 3′ adapter and a 5′ adapter. Subsequently, reverse transcription primers were used to convert the ligated RNAs into cDNAs. The resulting cDNAs were then amplified through polymerase chain reaction (PCR) and purified via gel electrophoresis. The quality of the cDNAs was assessed using an Agilent 2100 chip (Agilent, Santa Clara, USA). For the analysis of the RNA-seq library, which focused on mRNAs and lncRNAs, the total RNA was purified to eliminate rRNA with the Ribo-Zero™ rRNA Removal kit (Epicentre-Illumina, Madison, USA), followed by fragmentation of the RNA. The fragmented RNA was then converted into first-strand cDNA using a TruSeq® Stranded Kit (Epicentre-Illumina). Double-stranded cDNA was generated using DNA polymerase I and RNase H. The 3′ ends of the double-stranded cDNA were adenylated and then ligated with adapters. PCR amplification and purification were carried out to construct the cDNA library. Sequencing of the libraries was conducted using the Illumina HiSeq 2500 platform for total RNA sequencing, employing a 90-bp paired-end sequencing strategy, while small RNA sequencing was performed on the Illumina HiSeq X Ten platform.

### Transmission electron microscopy (TEM)

For TEM observation of human placental tissues, sections measuring approximately 1 mm × 1 mm × 1 mm were prepared. The tissues and cells were then fixed using fixative buffer containing 2% paraformaldehyde and 2.5% glutaraldehyde in 0.1 M PBS. After being embedded, the tissues were sliced into 0.12-μm-thick sections and subsequently stained with 2% uranyl acetate and 4% lead citrate. Visual examination of the placentas was performed using a Zeiss TEM instrument (Zeiss, Oberkochen, Germany).

### Immunofluorescence staining

Human placental tissue sections were fixed using 4% paraformaldehyde (Solarbio) and permeabilized with 0.2% Triton X-100 (Solarbio). Subsequently, the sections were blocked with 5% goat serum for 30 minutes at room temperature. Primary antibodies against LC3B (1:100, ab192890; Abcam, Cambridge, UK), p62 (1:100, ab207305; Abcam) or CK-7 (1:100, ab216016; Abcam) were then added, and the sections were incubated overnight at 4°C. After wash with PBS, the sections were incubated with Alexa Fluor 488 (1:200, ab150113; Abcam) or Alexa Fluor 647 (1:200, ab150079; Abcam) secondary antibodies for 1 h at 37°C. Subsequently, the sections were washed with PBS, and the nuclei were stained with 4′,6-diamidino-2-phenylindole (DAPI) for 8 min in the dark. The fluorescence images were captured using a confocal microscope (Olympus, Tokyo, Japan).

### Immunohistochemistry (IHC)

The placental tissues were fixed overnight in a 4% formalin solution and subsequently embedded in paraffin using standard procedures. Five-micron-thick transverse sections were then obtained from the embedded tissues. Immunostaining was then carried out using antibodies against LC3 and p62 (1:200; Abcam). First, the sections were incubated with 5% inactivated goat serum for 1 h at room temperature to block nonspecific binding sites. The primary antibodies were then applied to the sections, which were then incubated overnight at 4°C. After thorough washing, the secondary antibodies including goat anti-rat IgG H&L (Alexa Fluor® 488) (ab150157; Abcam) and goat anti-rabbit IgG H&L (Alexa Fluor® 568) (ab175471; Abcam) were applied to the sections, which were then incubated for 2 h at room temperature. To visualize the staining, a diaminobenzidine (DAB) solution was used, resulting in a brown color. To visualize the nuclei, hematoxylin counterstaining was performed. The staining results were examined, and images were captured using an optical microscope (Olympus).

### Analysis of autophagic flux

Autophagic flux was assessed using mRFP-GFP-LC3 adenovirus vectors (HanBio, Shanghai, China). HTR8/SVneo and JEG-3 cells were transfected with the vectors using Lipofectamine 2000 (Invitrogen, Carlsbad, USA), followed by visualization of GFP and mRFP expression using a laser scanning confocal microscope (Olympus). Autophagosomes were identified as yellow puncta (mRFP ^+^ and GFP ^+^), while autolysosomes were identified as red puncta (mRFP ^+^ and GFP ^−^). The relative ratio of red/yellow puncta served as an indicator of autophagic flux.

### Western blot analysis

A Whole Protein Extraction kit (KeyGEN, Nanjing, China) was used for the extraction of cellular proteins, and the protein concentration was subsequently determined with a BCA Protein Assay kit (KeyGEN). The proteins were separated by 12% sodium dodecylulfate-polyacrylamide gel electrophoresis (SDS-PAGE) and subsequently transferred onto nitrocellulose membranes. Following a 2-h blocking period with 5% nonfat milk, the membranes were incubated overnight at 4°C with the appropriate primary antibodies. These primary antibodies included antibodies against LC3B and p62 (Abcam), which were diluted at a ratio of 1:1000. The membranes were washed three times before being incubated with horseradish peroxidase (HRP)-conjugated secondary antibodies for 2 h. Subsequently, a chemiluminescence kit (KeyGEN) was used to detect protein expression. The optical density of the bands was quantified using densitometry and then normalized to that of the β-actin loading control.

### Statistical analysis

Statistical analysis was conducted using GraphPad Prism 7.0 (GraphPad Software, La Jolla, USA). Data are expressed as the mean ± SD and were derived from a minimum of three independent experiments. Differences between groups were assessed using one-way analysis of variance, followed by the Student-Newman-Keuls post hoc test or the nonparametric Mann-Whitney U test. *P* < 0.05 was considered to indicate statistical significance.

## Results

### Clinical baseline characteristics of the included subjects

We collected 85 placentas from patients who underwent surgical termination of pregnancy. The clinicopathological characteristics of the 85 pregnancies are presented in Table 1. There was no significant difference in maternal age or fetal sex between healthy pregnancies and preeclamptic pregnancies ( *P* > 0.05). However, blood pressure (systolic and diastolic) and proteinuria were significantly greater in pregnant patients with PE ( *P* < 0.0001). In addition, there was a significant difference in maternal body mass index (BMI) between healthy pregnancies and preeclamptic pregnancies ( *P* < 0.05). Moreover, preeclamptic pregnancies had a decreased gestational age at delivery and neonatal birth weight compared with healthy pregnancies ( *P* < 0.05), indicating that these clinicopathological parameters were in accordance with the diagnostic criteria.

**Table:** **Table 1** Clinical data of healthy and preeclamptic pregnancies

Characteristic	PC ( *n* = 40)	PE ( *n* = 45)	*P*-value
Maternal age (years)	28.13 ± 4.28	27.49 ± 4.63	0.4818
Gestational age (weeks)	39.57 ± 1.11	38.43 ± 2.05	0.0022
BMI (kg/m ^2^)	28.62 ± 0.57	30.44 ± 0.63	0.0374
Systolic blood pressure (mmHg)	113.00 ± 1.53	145.00 ± 2.18	< 0.0001
Diastolic blood pressure (mmHg)	73.00 ± 1.20	97.00 ± 1.30	< 0.0001
Urine protein/24 h (g)	N/A	2.86 ± 0.31	N/A
Neonatal birth weight (g)	3384.00 ± 65.10	3049.00 ± 106.80	0.0111
Fetal gender (male/female)	17/23	26/19	0.3316

### Microarray analysis of differentially expressed genes in placentas from preeclamptic pregnancies and healthy pregnancies

**Figure 1:**
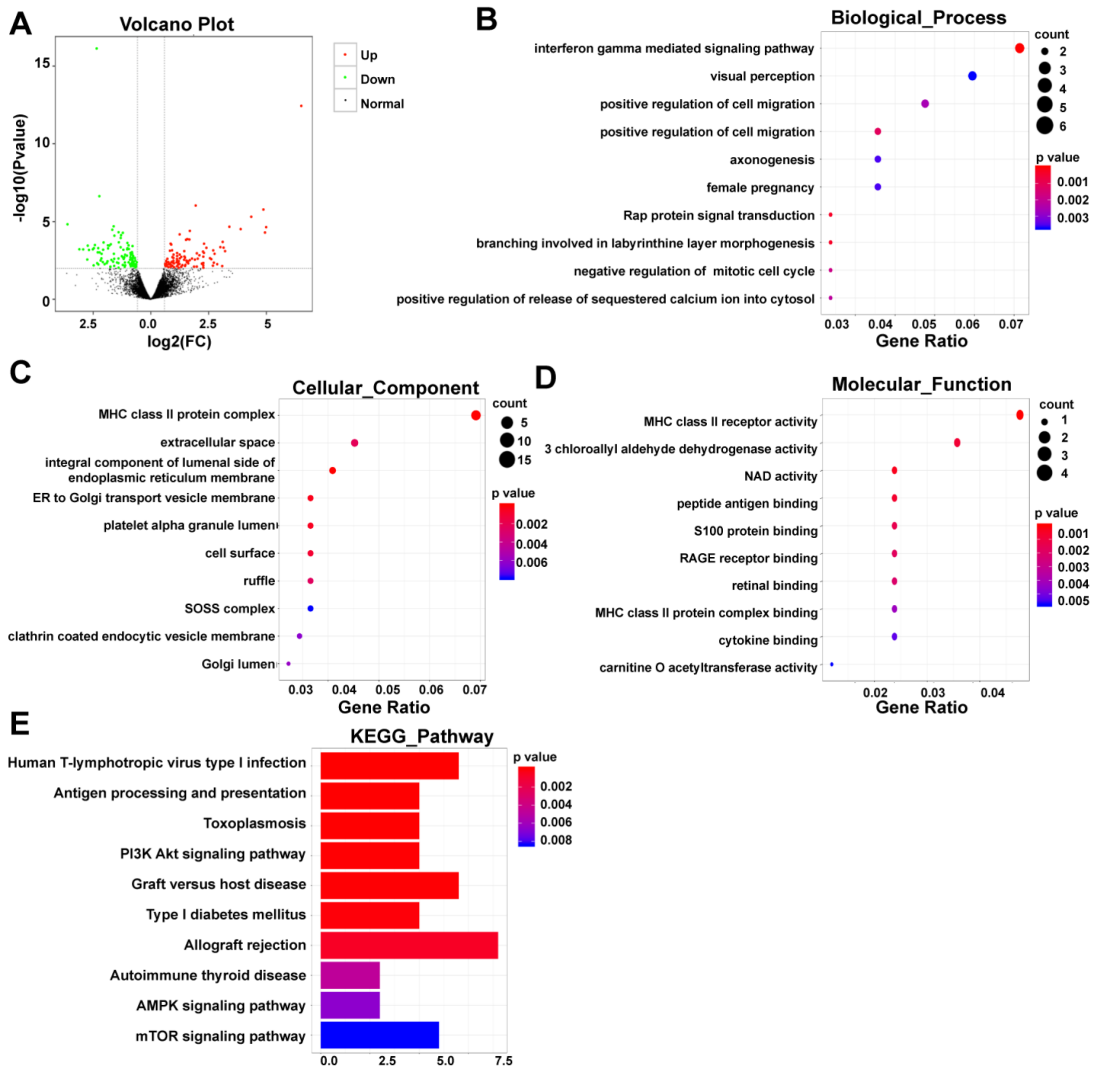
Figure 1 Identification of differentially expressed genes in placentas from preeclamptic pregnancies and healthy pregnancies (A) Volcano plot showing clustering of differentially expressed genes (DEGs) in placentas from healthy pregnancies and preeclamptic pregnancies. (B‒D) Functional predictions of the top 10 enriched biological process (BP), cellular component (CC) and molecular function (MF) terms determined by Gene Ontology (GO) analysis. (E) KEGG signaling pathway histogram. The ordinate on the left represents top 10 KEGG signaling pathways.

Placental dysfunction has been widely recognized as the primary etiology of PE [14]. To investigate the differential expression of genes (DEGs) between preeclamptic pregnancy and healthy pregnancy, RNA-seq analysis was conducted on placental tissues. The differential expression analysis utilizing DeSeq2 and nNOR algorithms identified a total of 218 genes ( Supplementary Table S1) with significant changes (fold change ≥ 2.0, *P* ≤ 0.05) in placentas from preeclamptic pregnancies. Among these genes, 113 were upregulated, while 105 were downregulated ( Figure 1A). In order to explore the biological functions of the DEGs, we leveraged the capabilities of R software to conduct GO analysis and KEGG pathway enrichment analysis. The GO framework comprises three categories, namely biological process, cellular component, and molecular function. These categories aid in understanding the alterations in biological functions associated with placentas affected by PE ( Figure 1B‒D). The KEGG pathway analysis demonstrated that the DEGs were primarily associated with several pathways, including Human T-lymphotropic virus type I infection, Antigen processing and presentation, and Toxoplasmosis. Remarkably, we observed a significant enrichment of the DEGs in pathways such as PI3K-Akt signaling, AMPK signaling, and mTOR signaling, which have been extensively linked to the regulation of autophagy ( Figure 1E). These findings indicate that autophagy may have a significant impact on the development of PE, potentially involving these genes ( Supplementary Table S2).

### Autophagy is increased in placentas from preeclamptic pregnancies

**Figure 2:**
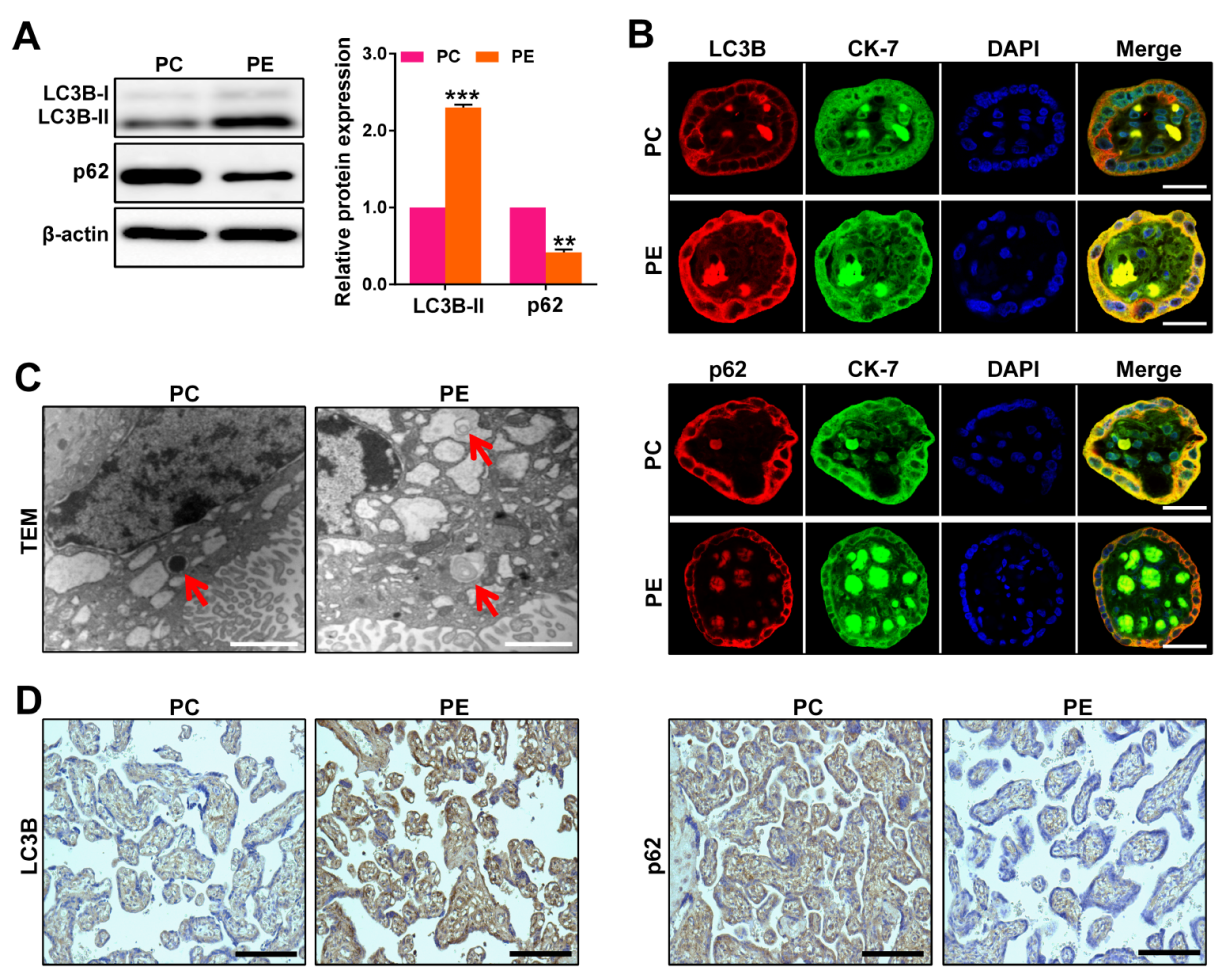
Figure 2 Autophagy is increased in placentas from preeclamptic pregnancies (A) Western blot analysis of LC3B-II and p62 expressions in placentas from healthy pregnancies and preeclamptic pregnancies. (B) Colocalization of LC3B (red) or p62 (red) with cytokeratin-7 (CK-7, a trophoblast-specific marker, green) in placentas from preeclamptic pregnancies and healthy pregnancies, respectively. The nuclei were stained with DAPI (blue). Scale bar: 50 μm. (C) Representative transmission electron microscopy (TEM) images of placentas from preeclamptic pregnancies and healthy pregnancies. Autophagosomes are indicated with red arrows. Scale bar: 1000 nm. (D) Representative immunohistochemical staining of LC3B and p62 in placentas from preeclamptic pregnancies and healthy pregnancies. Scale bar: 200 μm. Data are presented as the mean ± SD. ** P < 0.01, ***P < 0.001.

To evaluate the effect of preeclampsia on autophagy levels in placentas, we conducted western blot analysis to measure the expressions of LC3B-II and p62, which are well-established markers of autophagy. Figure 2A illustrates that LC3B-II expression was significantly upregulated, while p62 expression was downregulated in placentas from preeclamptic pregnancies. Additionally, we performed immunofluorescence staining with the trophoblast-specific marker CK-7 to investigate the expressions of LC3B and p62 in the placenta. As depicted in Figure 2B, trophoblasts in placentas from preeclamptic pregnancies exhibited elevated level of LC3B and reduced level of p62. Moreover, TEM results revealed that the number of autophagosomes or autolysosomes was increased in the placenta of preeclampsia patients ( Figure 2C). Furthermore, we employed immunohistochemical staining to examine the expressions of LC3B and p62 in the placentas. As shown in Figure 2D, there was a noticeable increase in LC3B expression and a decrease in p62 expression in placentas from preeclamptic pregnancies. Overall, our results strongly suggest that autophagy is significantly enhanced in placentas affected by preeclampsia.

### Autophagy is increased in trophoblasts under hypoxia.

**Figure 3:**
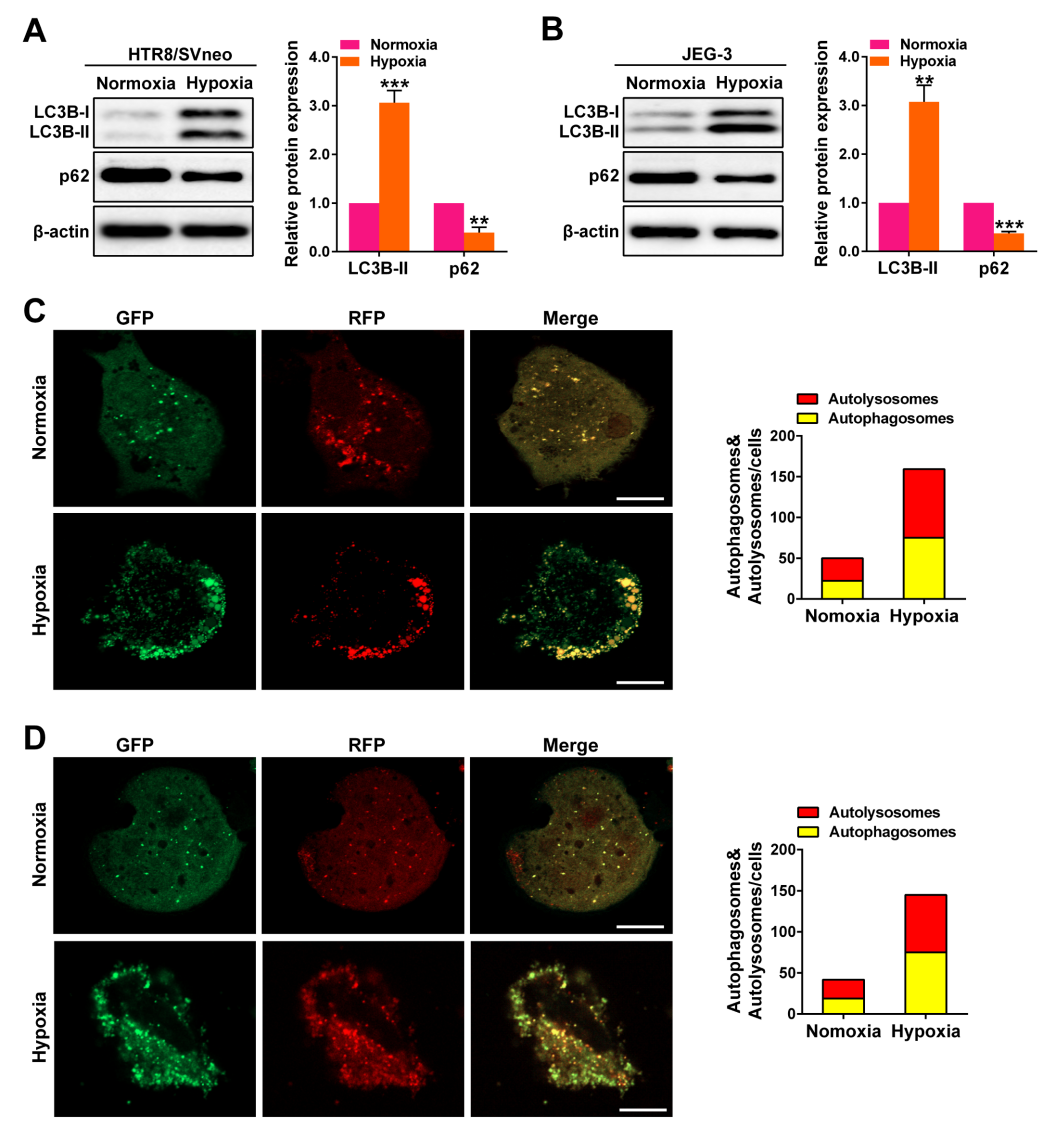
Figure 3 Autophagy is increased in trophoblasts under hypoxia (A,B) Western blot analysis of LC3B-II and p62 expressions in HTR8/SVneo and JEG-3 cells under hypoxia. (C,D) Fluorescence images of mRFP-GFP-LC3 in HTR8/SVneo and JEG-3 cells under hypoxia were obtained by laser confocal microscopy. Scale bar: 20 μm. Data are presented as the mean ± SD. **P < 0.01, *** P < 0.001.

Hypoxia plays a significant role in the development of pathological conditions in preeclampsia. To investigate how hypoxia affects placental cell lines, we conducted experiments using HTR8/SVneo and JEG-3 cells cultured under normoxic and hypoxic conditions. Our results revealed that the expression of LC3B-II, a key indicator of autophagy, increased in both cell lines under hypoxic conditions, mirroring observations in preeclamptic pregnancies. In contrast, the expression of p62, a protein involved in autophagic degradation, displayed the opposite pattern ( Figure 3A,B). To further evaluate the autophagic activity in these cells, we utilized mRFP-GFP-LC3 adenoviral vectors, which enable visualization and characterization of autophagic structures. After transfection of HTR8/SVneo and JEG-3 cells, we observed a dense accumulation of mRFP-LC3 and GFP-LC3 puncta in the perinuclear region and cytoplasm under hypoxic conditions ( Figure 3C,D).

### Autophagy inhibition alleviates preeclampsia-like symptoms

**Figure 4:**
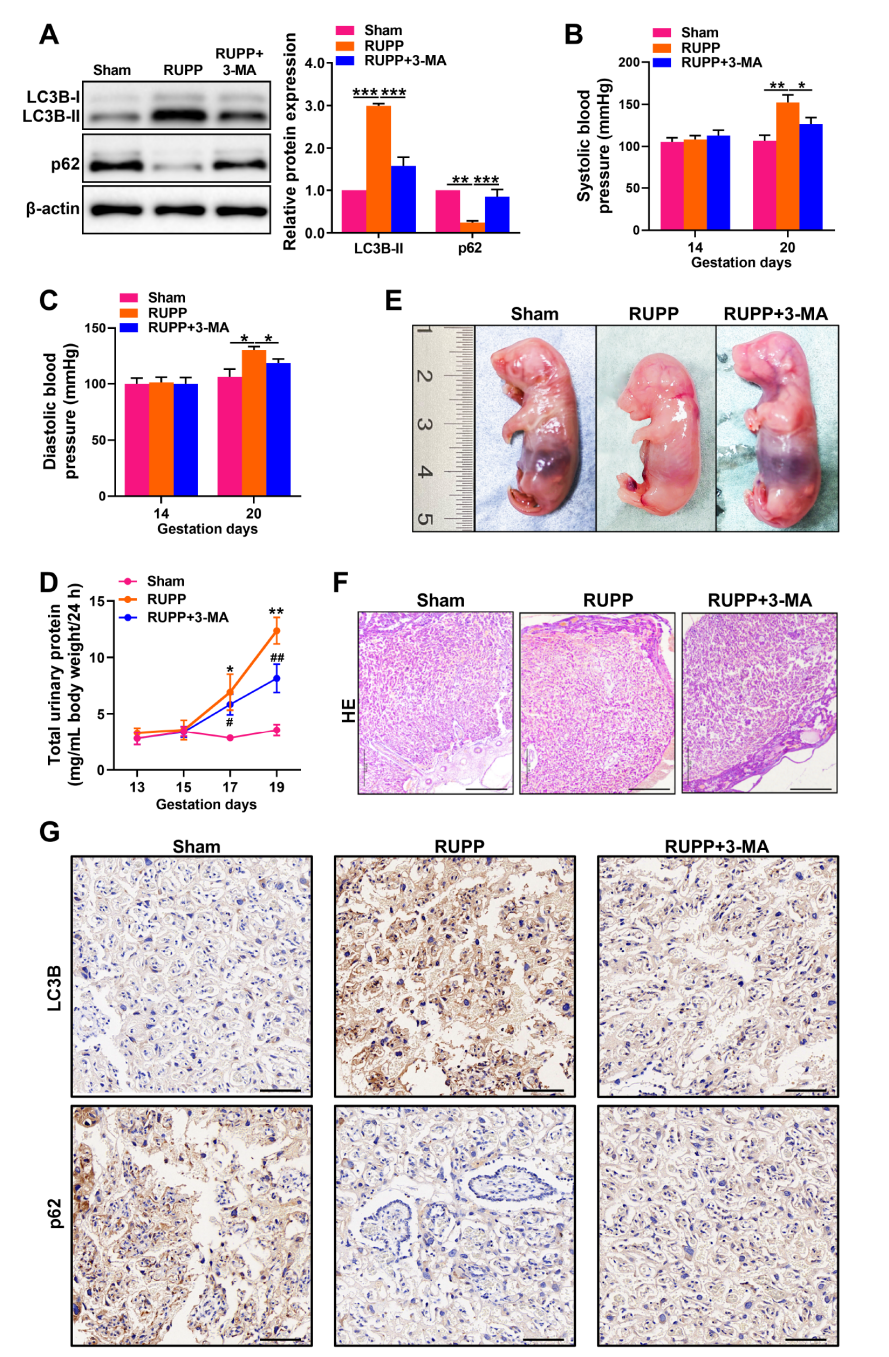
Figure 4 The inhibition of autophagy alleviates PE-like symptoms (A) Western blot analysis of LC3B-II and p62 expressions in the placentas of preeclamptic rats treated with 3-MA. (B,C) The systolic blood pressure and diastolic blood pressure of preeclamptic rats treated with 3-MA were measured with a noninvasive tail-cuff blood pressure measurement system. (D) A protein assay was used to detect total urine protein levels in preeclamptic rats treated with 3-MA. (E) Gross appearance of the foetus at embryonic day 18.5 (E18.5). (F) Placental pathological changes in a preeclamptic rat model induced by reduced uterine perfusion pressure (RUPP) combined with 3-MA treatment were determined by HE staining. Scale bar: 500 μm. (G) Immunohistochemical staining of LC3B and p62 in placentas from preeclamptic rats injected with 3-MA. Scale bar: 200 μm. Data are presented as the mean ± SD. *P < 0.05, ** P < 0.01, ***P < 0.001 vs sham; # P < 0.05, ##P < 0.01 vs RUPP.

To better understand the role of autophagy in the development of placental dysfunction in PE, we used a rat model of PE to assess the impact of treatment with 3-MA, a specific inhibitor of autophagy, on autophagy. Our aim was to explore the connection between autophagy and the progression of PE by examining the effect of 3-MA on autophagy in this animal model. The findings demonstrated that administration of 3-MA resulted in a significant reduction in the expression of LC3B-II and a significant increase in the expression of p62 ( Figure 4A). These results indicate that 3-MA effectively suppresses autophagy.

In addition, 3-MA treatment reduced the severity of PE-like symptoms in rats treated with RUPP, as indicated by a reduction in systolic blood pressure ( Figure 4B), diastolic blood pressure ( Figure 4C), and catabatic proteinuria ( Figure 4D). Additionally, the weight of the fetuses decreased in the rats, which was relieved by 3-MA ( Figure 4E). HE staining analysis demonstrated that treatment with 3-MA effectively attenuated the hydropic degeneration of decidual cells and reduced fibrous protein deposition in RUPP-treated rats ( Figure 4F). Additionally, immunohistochemical staining also confirmed that the expression of LC3B was significantly reduced, while p62 expression was enhanced in placentas from preeclamptic rats injected with 3-MA ( Figure 4G). These results collectively indicate that the autophagy inhibitor 3-MA has a beneficial impact on the development of PE in a rat model.

## Discussion

PE is a severe pregnancy-related hypertensive disorder that affects approximately 5%–8% of pregnancies worldwide. It poses a significant risk to the health of both mothers and unborn children, leading to substantial morbidity and mortality [ 15, 16]. PE is characterized by the onset of high blood pressure after 20 weeks of gestation and is accompanied by proteinuria, organ damage, and abnormal placental development [17]. Although the exact cause of PE remains unknown, it is widely acknowledged that its pathogenesis originates from the placenta, where alterations in trophoblast function result in inadequate placental perfusion [18]. Therefore, gaining a comprehensive understanding of the mechanisms underlying placental trophoblast dysfunction is highly important for the prevention and treatment of PE.

Autophagy is a vital cellular process that responds to environmental stressors, including hypoxia, undernutrition, and inflammation [ 19, 20]. It serves as an adaptive mechanism to maintain cellular energy balance by degrading long-lived proteins and eliminating damaged organelles and misfolded proteins through the autolysosomal degradation pathway [21]. Recent studies support the hypothesis that autophagic dysfunction is associated with preeclampsia [ 22‒ 24]. Dysregulation of autophagy may induce placental dysfunction by preventing homeostasis maintenance [ 23, 25, 26] and consequently influencing multiple cell behaviors, including trophoblast invasion, in the process of human placentation [ 25‒ 28]. Zhao *et al* . [29] reported that PKCβ contributes to the pathogenesis of preeclampsia through autophagy-mediated impairment of placental angiogenesis. In addition, Yang *et al*. [30] demonstrated that the lncRNA SNHG5 acts as a sponge for miR-31-5p, thereby promoting SPARC transcription, alleviating the PE phenotype, and inhibiting trophoblastic autophagy. In our study, we examined transcriptomic and pathway data from placentas of preeclamptic pregnancies and healthy pregnancies. Our analysis revealed significant enrichment of three autophagy-related signaling pathways, namely, the PI3K-Akt, AMPK, and mTOR signaling pathways, in preeclamptic placentas. Subsequent experimental evidence confirmed the activation of autophagy in preeclamptic placentas, as evidenced by increased level of the autophagy marker LC3 and decreased level of p62, as well as the presence of autophagolysosomes. In addition, our *in vitro* experiments demonstrated the activation of autophagy in JEG-3 cells and HTR-8/SVneo cells under hypoxic conditions. These findings suggest that increased autophagy plays a crucial role in contributing to placental dysfunction. One specific autophagy inhibitor, 3-MA, is a group of PI3K inhibitors that hinder autophagy at the initiation and maturation stages. This inhibition is achieved by impeding the interaction between class III PI3K and various ATG partners [31]. Currently, the use of 3-MA to inhibit autophagy shows promise as a potential therapeutic approach in animal models of cancer. For example, 3-MA was found to inhibit autophagy and excessive activity in microglia, leading to the transformation of microglia from the M1 to the M2 subtype. This transformation promotes the recovery of brain tissue after exposure to radiation in a rat model [32]. Moreover, pretreatment with 3-MA was found to alleviate acute pneumonia induced by *Pseudomonas aeruginosa* in a mouse infection model by inhibiting neutrophil death [33]. The impact of inhibiting autophagy through 3-MA on PE has not been fully explored. Thus, we investigated the effects of 3-MA-mediated autophagy inhibition on PE in rats. Our findings revealed that 3-MA treatment led to a decrease in LC-3 II expression and an increase in p62 expression, indicating suppressed autophagy in the rats. Importantly, we observed a significant reduction in the severity of PE-like symptoms in rats subjected to RUPP and treated with 3-MA. This reduction was evident in various parameters, including systolic and diastolic blood pressure, catabatic proteinuria, and fetal weight. Our data imply that 3-MA has the potential to alleviate excessive placental autophagy during PE development and improve this condition.

**Figure 5:**
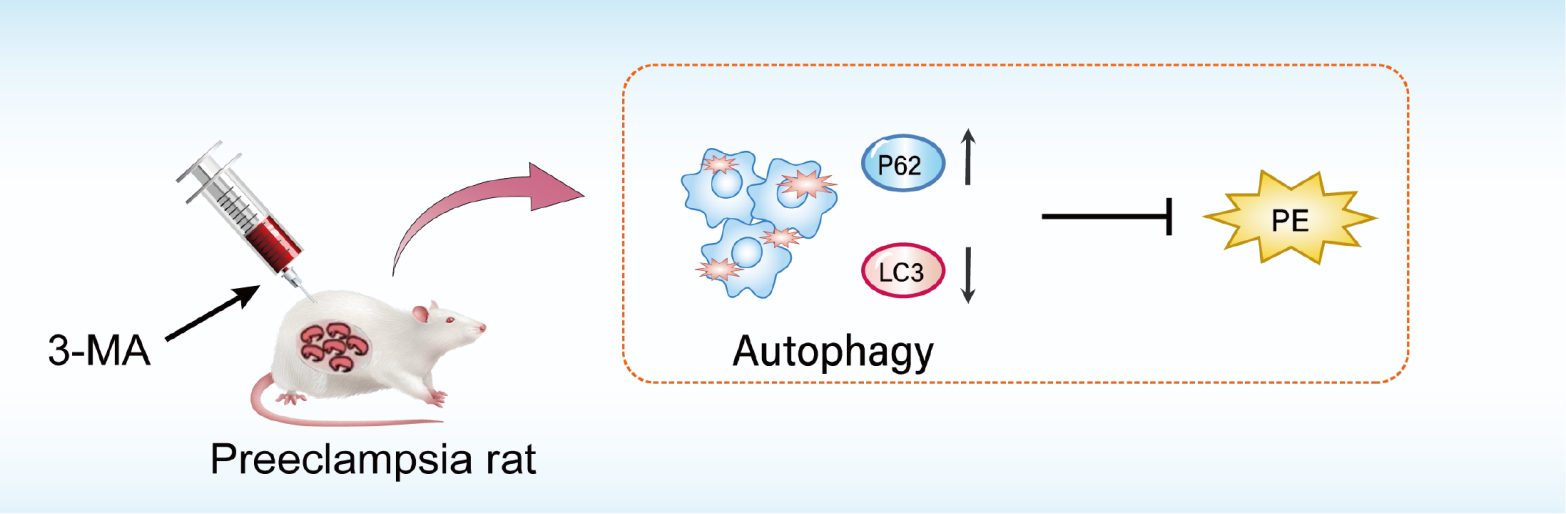
Figure 5 Schematic diagram showing the mechanism by which 3-MA inhibits autophagy in preeclampsia The autophagy inhibitor 3-MA inhibits the expressions of autophagy proteins in the development of preeclampsia.

In summary, our findings elucidated the beneficial effects of 3-MA treatment in alleviating symptoms of PE in rats, thereby offering valuable insights into the development of pharmacological interventions for PE prevention ( Figure 5). Despite the promising therapeutic potential of 3-MA, further investigation is necessary to gain a comprehensive understanding of the underlying mechanisms involved.

## Supporting information

24039Supplement_table2

24039Supplement_table_1

## Supplementary Data

Supplementary data is available at *Acta Biochimica et Biophysica Sinica* online.
