# Supplementary material for: Inhibition of autophagy via 3-methyladenine alleviates the progression of preeclampsia: 3-Methyladenine alleviates the progression of preeclampsia
Source: Acta Biochim Biophys Sin (Shanghai). 2024 Jul 8;57(3):356–64. doi: 10.3724/abbs.2024096 (PMC11986455; doi:10.3724/abbs.2024096)
Supplement: 24039Supplement_table2 [file 24039Supplement_table2.docx]

**Supplementary Table S2. List of differential expression of autophagy related genes in transcriptomic data**

| #ID | Symbol | *P* value | log2FC | regulated |
| --- | --- | --- | --- | --- |
| ENSG00000104689 | TNFRSF10A | 0.029892021 | -8.506056147 | down |
| ENSG00000175294 | CATSPER1 | 0.029734591 | -8.407426447 | down |
| ENSG00000179921 | GPBAR1 | 0.017908612 | -8.311288238 | down |
| ENSG00000150455 | TIRAP | 0.000776001 | -8.094508956 | down |
| ENSG00000185591 | SP1 | 0.022860327 | -7.793566129 | down |
| ENSG00000100030 | MAPK1 | 0.038130133 | -7.39168121 | down |
| ENSG00000135914 | HTR2B | 0.049298485 | -6.599672947 | down |
| ENSG00000143514 | TP53BP2 | 0.005156158 | 9.199609239 | up |
| ENSG00000204397 | CARD16 | 0.011405839 | 9.1450896 | up |
| ENSG00000112079 | STK38 | 0.003393887 | 7.804789036 | up |
| ENSG00000115350 | POLE4 | 0.030301782 | 7.213891963 | up |
| ENSG00000117595 | IRF6 | 0.020123847 | 6.733729144 | up |
| ENSG00000181652 | ATG9B | 0.031506337 | 3.596512226 | up |
| Human_newGene_92609 | Human_newGene_92609 | 0.005894202 | 3.590196963 | up |
| ENSG00000168010 | ATG16L2 | 0.020144052 | 2.52620347 | up |
