# Supplementary material for: Inhibition of autophagy via 3-methyladenine alleviates the progression of preeclampsia: 3-Methyladenine alleviates the progression of preeclampsia
Source: Acta Biochim Biophys Sin (Shanghai). 2024 Jul 8;57(3):356–64. doi: 10.3724/abbs.2024096 (PMC11986455; doi:10.3724/abbs.2024096)
Supplement: 24039Supplement_table_1 [file 24039Supplement_table_1.docx]

**Supplementary Table S1. List of differentially expressed genes**

| #ID | Symbol | *P* value | log2FC | Regulated |
| --- | --- | --- | --- | --- |
| ENSG00000243543 | WFDC6 | 0.021357421 | -8.74544784 | down |
| ENSG00000168418 | KCNG4 | 0.039432785 | -8.638484727 | down |
| ENSG00000104689 | TNFRSF10A | 0.029892021 | -8.506056147 | down |
| ENSG00000175294 | CATSPER1 | 0.029734591 | -8.407426447 | down |
| ENSG00000179921 | GPBAR1 | 0.017908612 | -8.311288238 | down |
| ENSG00000177873 | ZNF619 | 0.000531563 | -8.309663223 | down |
| ENSG00000139133 | ALG10 | 0.021800206 | -8.223919445 | down |
| ENSG00000150455 | TIRAP | 0.000776001 | -8.094508956 | down |
| ENSG00000082805 | ERC1 | 0.012332026 | -8.021235225 | down |
| ENSG00000185591 | SP1 | 0.022860327 | -7.793566129 | down |
| ENSG00000070778 | PTPN21 | 0.010141589 | -7.455016196 | down |
| ENSG00000100030 | MAPK1 | 0.038130133 | -7.39168121 | down |
| ENSG00000196639 | HRH1 | 0.028516778 | -7.358666907 | down |
| ENSG00000175938 | ORAI3 | 0.033869703 | -7.272444549 | down |
| ENSG00000006042 | TMEM98 | 0.01774954 | -7.265008924 | down |
| ENSG00000116962 | NID1 | 0.030888924 | -7.167817386 | down |
| ENSG00000174807 | CD248 | 0.011920157 | -6.980276116 | down |
| ENSG00000103187 | COTL1 | 0.014843687 | -6.970941803 | down |
| ENSG00000135119 | RNFT2 | 0.037064022 | -6.94114296 | down |
| ENSG00000131238 | PPT1 | 0.003366576 | -6.938184171 | down |
| ENSG00000196502 | SULT1A1 | 0.037938124 | -6.921796708 | down |
| ENSG00000116525 | TRIM62 | 0.004563273 | -6.921397053 | down |
| ENSG00000140092 | FBLN5 | 0.014342641 | -6.920602288 | down |
| ENSG00000171812 | COL8A2 | 0.032081071 | -6.916460836 | down |
| Human_newGene_189061 | Human_newGene_189061 | 0.024559876 | -6.914773625 | down |
| ENSG00000176532 | PRR15 | 0.023225299 | -6.913060115 | down |
| ENSG00000107518 | ATRNL1 | 0.019523328 | -6.902689702 | down |
| ENSG00000239887 | C1orf226 | 0.024302573 | -6.808454354 | down |
| ENSG00000174099 | MSRB3 | 0.019473774 | -6.803310425 | down |
| ENSG00000138613 | APH1B | 0.043306604 | -6.795825065 | down |
| ENSG00000149090 | PAMR1 | 0.003517018 | -6.794064058 | down |
| ENSG00000134548 | SPX | 0.047554421 | -6.673319517 | down |
| ENSG00000087237 | CETP | 0.041350513 | -6.625273864 | down |
| ENSG00000135914 | HTR2B | 0.049298485 | -6.599672947 | down |
| Human_newGene_242370 | Human_newGene_242370 | 0.007957222 | -6.536290634 | down |
| ENSG00000114251 | WNT5A | 0.002677899 | -6.334718976 | down |
| ENSG00000197852 | INKA2 | 0.033646889 | -6.327665855 | down |
| ENSG00000177098 | SCN4B | 0.032374401 | -6.289890772 | down |
| Human_newGene_174310 | Human_newGene_174310 | 0.0443322 | -6.270045476 | down |
| ENSG00000130988 | RGN | 0.022175893 | -6.209463143 | down |
| ENSG00000187479 | C11orf96 | 0.017705467 | -6.161891064 | down |
| Human_newGene_91003 | Human_newGene_91003 | 0.024278227 | -6.095445994 | down |
| ENSG00000128045 | RASL11B | 0.011305295 | -5.630033245 | down |
| ENSG00000173366 | AC097637.1 | 0.008785849 | -5.620355593 | down |
| ENSG00000267303 | AC011511.4 | 0.016184448 | -5.499399857 | down |
| ENSG00000188778 | ADRB3 | 0.010434886 | -5.220966551 | down |
| ENSG00000150594 | ADRA2A | 0.000330132 | -5.199088841 | down |
| ENSG00000007908 | SELE | 0.043493704 | -5.173989363 | down |
| ENSG00000240694 | PNMA2 | 0.016957993 | -5.165227698 | down |
| ENSG00000171560 | FGA | 0.001383935 | -5.129447487 | down |
| ENSG00000100628 | ASB2 | 0.011131925 | -5.124660641 | down |
| ENSG00000170961 | HAS2 | 0.007741605 | -5.109986117 | down |
| ENSG00000008394 | MGST1 | 0.028658176 | -5.042629145 | down |
| ENSG00000158445 | KCNB1 | 0.038304405 | -5.034660546 | down |
| ENSG00000256407 | AL357673.1 | 0.031982724 | -4.93538468 | down |
| ENSG00000196562 | SULF2 | 0.038394124 | -4.639222055 | down |
| ENSG00000184731 | FAM110C | 0.040029597 | -4.550363395 | down |
| Human_newGene_7268 | Human_newGene_7268 | 0.035017412 | -4.54612948 | down |
| ENSG00000047936 | ROS1 | 0.020689077 | -4.464406524 | down |
| ENSG00000154975 | CA10 | 0.000787512 | -4.43347915 | down |
| ENSG00000128918 | ALDH1A2 | 0.046464323 | -4.426496154 | down |
| ENSG00000178301 | AQP11 | 0.007329871 | -4.412322374 | down |
| ENSG00000115523 | GNLY | 0.046954807 | -4.353746384 | down |
| ENSG00000276966 | HIST1H4E | 0.001339049 | -4.336303834 | down |
| ENSG00000184227 | ACOT1 | 0.035189563 | -4.333194704 | down |
| ENSG00000124766 | SOX4 | 0.00937304 | -4.329872718 | down |
| ENSG00000239839 | DEFA3 | 0.018671129 | -4.321479648 | down |
| ENSG00000198794 | SCAMP5 | 0.037206271 | -4.283017176 | down |
| ENSG00000118785 | SPP1 | 0.028320684 | -4.274771016 | down |
| ENSG00000124479 | NDP | 0.01204645 | -3.969923173 | down |
| ENSG00000086289 | EPDR1 | 0.012482159 | -3.689289183 | down |
| ENSG00000141905 | NFIC | 0.015053486 | -3.652404654 | down |
| ENSG00000198797 | BRINP2 | 0.016361985 | -3.649586364 | down |
| ENSG00000162493 | PDPN | 0.009681626 | -3.649355779 | down |
| ENSG00000105989 | WNT2 | 0.005816015 | -3.649112836 | down |
| ENSG00000006016 | CRLF1 | 0.00658288 | -3.630839733 | down |
| ENSG00000221869 | CEBPD | 0.001553304 | -3.629745374 | down |
| ENSG00000165655 | ZNF503 | 0.041353746 | -3.613045664 | down |
| ENSG00000061918 | GUCY1B1 | 0.00836944 | -3.612345431 | down |
| ENSG00000141404 | GNAL | 0.01546101 | -3.597079601 | down |
| ENSG00000267059 | AC005943.1 | 0.029961605 | -3.47047091 | down |
| Human_newGene_32218 | Human_newGene_32218 | 0.044638894 | -3.403633514 | down |
| ENSG00000171557 | FGG | 0.013225938 | -3.289693181 | down |
| Human_newGene_189497 | Human_newGene_189497 | 0.008139684 | -3.155862724 | down |
| ENSG00000171564 | FGB | 0.004555414 | -3.092408616 | down |
| ENSG00000105696 | TMEM59L | 0.020561146 | -2.982764838 | down |
| ENSG00000172179 | PRL | 0.03167225 | -2.972721975 | down |
| ENSG00000180834 | MAP6D1 | 0.010773443 | -2.92837991 | down |
| ENSG00000089116 | LHX5 | 0.014954311 | -2.918632508 | down |
| Human_newGene_267722 | Human_newGene_267722 | 0.023272804 | -2.908175024 | down |
| Human_newGene_200387 | Human_newGene_200387 | 0.042538043 | -2.891342562 | down |
| Human_newGene_7454 | Human_newGene_7454 | 0.015462028 | -2.880712888 | down |
| ENSG00000146678 | IGFBP1 | 0.044883755 | -2.873273747 | down |
| ENSG00000163377 | FAM19A4 | 0.032236234 | -2.85992781 | down |
| ENSG00000111700 | SLCO1B3 | 0.042125761 | -2.784459615 | down |
| Human_newGene_48397 | Human_newGene_48397 | 0.017750456 | -2.743380509 | down |
| Human_newGene_237229 | Human_newGene_237229 | 0.025990529 | -2.731696028 | down |
| ENSG00000105426 | PTPRS | 0.006516145 | -2.455387499 | down |
| ENSG00000169105 | CHST14 | 0.014280845 | -2.399955299 | down |
| ENSG00000178033 | CALHM5 | 0.005706453 | -2.270847317 | down |
| ENSG00000187866 | FAM122A | 1.51E-10 | -2.219377846 | down |
| ENSG00000180785 | OR51E1 | 0.037126061 | -2.176253674 | down |
| ENSG00000167074 | TEF | 0.016169193 | -2.171005598 | down |
| ENSG00000205420 | KRT6A | 0.016438887 | -2.06411177 | down |
| ENSG00000103196 | CRISPLD2 | 0.020664738 | -2.000965216 | down |
| ENSG00000143514 | TP53BP2 | 0.005156158 | 9.199609239 | up |
| ENSG00000204397 | CARD16 | 0.011405839 | 9.1450896 | up |
| ENSG00000112079 | STK38 | 0.003393887 | 7.804789036 | up |
| ENSG00000171004 | HS6ST2 | 0.002138724 | 7.653932205 | up |
| ENSG00000066697 | MSANTD3 | 0.008041443 | 7.37900759 | up |
| ENSG00000115350 | POLE4 | 0.030301782 | 7.213891963 | up |
| ENSG00000169609 | C15orf40 | 0.002994637 | 7.118562421 | up |
| ENSG00000117595 | IRF6 | 0.020123847 | 6.733729144 | up |
| ENSG00000285446 | Z84488.2 | 0.017590376 | 6.724122523 | up |
| ENSG00000163599 | CTLA4 | 0.022369771 | 6.659983947 | up |
| ENSG00000164756 | SLC30A8 | 0.017601221 | 6.654802335 | up |
| ENSG00000163568 | AIM2 | 0.016442559 | 6.626074818 | up |
| ENSG00000197816 | CCDC180 | 0.00109709 | 6.530506317 | up |
| ENSG00000154529 | CNTNAP3B | 0.004482699 | 6.48881905 | up |
| ENSG00000100170 | SLC5A1 | 0.023307639 | 6.357405029 | up |
| ENSG00000258677 | AC022826.2 | 0.010251672 | 6.291027911 | up |
| ENSG00000089250 | NOS1 | 0.006688238 | 6.175933893 | up |
| ENSG00000124490 | CRISP2 | 0.001405564 | 6.047738959 | up |
| ENSG00000270316 | BORCS7-ASMT | 0.034246878 | 6.000076204 | up |
| ENSG00000183305 | MAGEA2B | 0.00054037 | 5.910485107 | up |
| ENSG00000254692 | AL136295.1 | 0.001602478 | 5.886052522 | up |
| ENSG00000148734 | NPFFR1 | 0.0041683 | 5.883621744 | up |
| ENSG00000161133 | USP41 | 0.035671905 | 5.711529934 | up |
| ENSG00000102794 | ACOD1 | 0.00358719 | 5.586288734 | up |
| ENSG00000277669 | AC009086.2 | 0.015208093 | 5.571199186 | up |
| ENSG00000255526 | NEDD8-MDP1 | 0.041126862 | 5.440273847 | up |
| ENSG00000272162 | AL024498.2 | 0.012744523 | 5.406178482 | up |
| ENSG00000138684 | IL21 | 0.001231658 | 5.375027297 | up |
| ENSG00000244255 | AL645922.1 | 0.018627018 | 5.358031774 | up |
| ENSG00000285396 | AC012476.1 | 0.043230253 | 5.33966456 | up |
| ENSG00000277957 | SENP3-EIF4A1 | 0.027574755 | 5.274939228 | up |
| ENSG00000105650 | PDE4C | 0.043443281 | 5.163506489 | up |
| ENSG00000284337 | AC013271.1 | 0.019538787 | 5.162222835 | up |
| ENSG00000240747 | KRBOX1 | 0.048733579 | 4.887073167 | up |
| ENSG00000284292 | AC004922.1 | 0.048590569 | 4.827117373 | up |
| ENSG00000242028 | HYPK | 0.019745652 | 4.657082915 | up |
| ENSG00000284431 | AL022238.4 | 0.006845915 | 4.634831765 | up |
| ENSG00000234857 | HNRNPUL2-BSCL2 | 0.009171994 | 4.538480373 | up |
| ENSG00000181009 | OR52N5 | 0.038878852 | 4.482298289 | up |
| ENSG00000197683 | KRTAP26-1 | 0.044135452 | 4.479909474 | up |
| ENSG00000113389 | NPR3 | 0.029978636 | 4.471015462 | up |
| ENSG00000147571 | CRH | 0.013305317 | 4.292867218 | up |
| Human_newGene_269464 | Human_newGene_269464 | 0.025093632 | 4.250068616 | up |
| Human_newGene_190079 | Human_newGene_190079 | 0.019189239 | 4.237979534 | up |
| Human_newGene_242211 | Human_newGene_242211 | 0.028620326 | 4.223370825 | up |
| Human_newGene_108986 | Human_newGene_108986 | 0.014227112 | 4.196732122 | up |
| Human_newGene_57019 | Human_newGene_57019 | 0.019253065 | 4.128140904 | up |
| Human_newGene_190250 | Human_newGene_190250 | 0.013406446 | 4.123927621 | up |
| Human_newGene_139584 | Human_newGene_139584 | 0.047502154 | 3.958678307 | up |
| Human_newGene_131750 | Human_newGene_131750 | 0.046806518 | 3.910668867 | up |
| Human_newGene_186145 | Human_newGene_186145 | 0.006778803 | 3.845689081 | up |
| ENSG00000198826 | ARHGAP11A | 0.00738972 | 3.756473436 | up |
| ENSG00000018280 | SLC11A1 | 0.00961454 | 3.618053995 | up |
| ENSG00000181652 | ATG9B | 0.031506337 | 3.596512226 | up |
| Human_newGene_92609 | Human_newGene_92609 | 0.005894202 | 3.590196963 | up |
| Human_newGene_235104 | Human_newGene_235104 | 0.023345456 | 3.560389797 | up |
| Human_newGene_83837 | Human_newGene_83837 | 0.008715583 | 3.49159434 | up |
| Human_newGene_189695 | Human_newGene_189695 | 0.039637548 | 3.469863516 | up |
| Human_newGene_254923 | Human_newGene_254923 | 0.016299307 | 3.319391404 | up |
| ENSG00000160336 | ZNF761 | 0.045194414 | 3.295927692 | up |
| ENSG00000214022 | REPIN1 | 0.006332278 | 3.184962246 | up |
| Human_newGene_218803 | Human_newGene_218803 | 2.62E-05 | 3.174213575 | up |
| Human_newGene_150432 | Human_newGene_150432 | 0.049955864 | 3.144777107 | up |
| Human_newGene_59330 | Human_newGene_59330 | 0.00177206 | 3.142025926 | up |
| Human_newGene_213513 | Human_newGene_213513 | 0.005192823 | 3.136229657 | up |
| ENSG00000181666 | HKR1 | 5.17E-05 | 2.963691277 | up |
| ENSG00000113916 | BCL6 | 0.042912478 | 2.93290483 | up |
| Human_newGene_54685 | Human_newGene_54685 | 0.037672927 | 2.92034223 | up |
| Human_newGene_31888 | Human_newGene_31888 | 0.012832294 | 2.906433006 | up |
| Human_newGene_245833 | Human_newGene_245833 | 0.038805139 | 2.874425289 | up |
| ENSG00000214021 | TTLL3 | 0.00912993 | 2.855197827 | up |
| ENSG00000149716 | LTO1 | 0.038502087 | 2.836118507 | up |
| ENSG00000008128 | CDK11A | 0.00123804 | 2.825436538 | up |
| ENSG00000155657 | TTN | 0.046862965 | 2.816788975 | up |
| ENSG00000173110 | HSPA6 | 0.005157478 | 2.796456908 | up |
| ENSG00000121716 | PILRB | 0.02801649 | 2.757880717 | up |
| ENSG00000132530 | XAF1 | 0.041268278 | 2.743704162 | up |
| Human_newGene_237114 | Human_newGene_237114 | 0.029580994 | 2.741556373 | up |
| Human_newGene_26394 | Human_newGene_26394 | 0.007422012 | 2.723013745 | up |
| ENSG00000114735 | HEMK1 | 0.044830374 | 2.704488958 | up |
| ENSG00000198642 | KLHL9 | 0.046121241 | 2.701568919 | up |
| Human_newGene_34731 | Human_newGene_34731 | 0.025410744 | 2.701471151 | up |
| ENSG00000138722 | MMRN1 | 0.041123311 | 2.699732133 | up |
| ENSG00000263956 | NBPF11 | 0.031294709 | 2.621752757 | up |
| ENSG00000243716 | NPIPB5 | 0.035845034 | 2.57381238 | up |
| ENSG00000243156 | MICAL3 | 2.05E-05 | 2.552428052 | up |
| ENSG00000214595 | EML6 | 0.005625364 | 2.550009629 | up |
| ENSG00000168010 | ATG16L2 | 0.020144052 | 2.52620347 | up |
| ENSG00000102287 | GABRE | 0.028701435 | 2.524841144 | up |
| ENSG00000173559 | NABP1 | 0.031347654 | 2.490908819 | up |
| Human_newGene_91245 | Human_newGene_91245 | 0.029645413 | 2.42980166 | up |
| ENSG00000196123 | KIAA0895L | 0.001100586 | 2.428223346 | up |
| ENSG00000131037 | EPS8L1 | 0.001697443 | 2.411380188 | up |
| ENSG00000266714 | MYO15B | 0.024895721 | 2.385123558 | up |
| ENSG00000104368 | PLAT | 0.011446862 | 2.380827634 | up |
| ENSG00000187627 | RGPD1 | 0.043121763 | 2.323014589 | up |
| ENSG00000158286 | RNF207 | 0.003411905 | 2.249644545 | up |
| Human_newGene_231766 | Human_newGene_231766 | 0.007498236 | 2.209852433 | up |
| ENSG00000137507 | LRRC32 | 0.011362203 | 2.18855912 | up |
| ENSG00000166801 | FAM111A | 0.001657136 | 2.187487329 | up |
| ENSG00000179532 | DNHD1 | 0.012225341 | 2.185611595 | up |
| ENSG00000160439 | RDH13 | 0.026308052 | 2.175882779 | up |
| ENSG00000101104 | PABPC1L | 0.000479308 | 2.136717462 | up |
| ENSG00000271425 | NBPF10 | 0.04710051 | 2.132182235 | up |
| ENSG00000204131 | NHSL2 | 0.029044989 | 2.113519107 | up |
| ENSG00000177943 | MAMDC4 | 0.008237852 | 2.098454112 | up |
| ENSG00000166473 | PKD1L2 | 0.018893399 | 2.096897527 | up |
| ENSG00000164308 | ERAP2 | 0.030787845 | 2.09681757 | up |
| ENSG00000269343 | ZNF587B | 0.020020752 | 2.044126692 | up |
| ENSG00000196912 | ANKRD36B | 0.00817383 | 2.037425377 | up |
| ENSG00000185864 | NPIPB4 | 0.001487991 | 2.034310049 | up |
| Human_newGene_218125 | Human_newGene_218125 | 0.004932374 | 2.016782783 | up |
| ENSG00000089916 | GPATCH2L | 0.030436528 | 2.00453453 | up |
